# Clinical and metagenomic characteristics of lymphadenopathy related to fever of unknown origin in children

**DOI:** 10.1038/s41390-024-03187-3

**Published:** 2024-04-27

**Authors:** Yajuan Zhou, Nan Shen, Lijuan Luo, Yandi Liu, Qing Cao

**Affiliations:** Department of Infectious Diseases, Shanghai Children’s Medical Center, School of Medicine, Shanghai Jiao Tong University, Shanghai, China

## Abstract

**Background:**

Diagnosis of fever of unknown origin remains challenge for pediatricians. Lymphadenopathy is a separate entity that mainly originates from infection or malignancy.

**Methods:**

168 patients with FUO accompanied by lymphadenectasis were reviewed. 33 lymph node tissue samples were examined by mNGS. Differences in clinical characteristics were compared among different disease groups. The value of mNGS in diagnosing and improving the clinical situation was assessed.

**Results:**

Multivariate analysis revealed that hepatosplenomegaly and LDH levels were associated with infectious diseases. Arthralgia was correlated with non-infectious inflammatory diseases. Weight loss and a node located in supraclavicular region may indicate neoplastic diseases. mNGS-positive rate was 60.60%, higher than that obtained with traditional methods. Treatment for 3/4 patients was adjusted according to the pathogen detected by mNGS, and antibiotics uses was discontinued or degraded in over 1/2 of the patients according to mNGS results.

**Conclusions:**

Clinical characteristics of children with lymphadenopathy related to FUO have limited diagnostic value for distinguishing different kinds of diseases, while mNGS of lymph node tissue serves as a useful tool for identifying infectious diseases, especially those caused by rare pathogens. mNGS results can lead to not only adjustments in targeted treatment but also further confirmation of underlying diseases.

**Impact Statement:**

1. The clinical features of children with FUO and lymphadenopathy differ according to disease group,although multivariate analysis indicated little diagnostic value for these features.
2. mNGS on lymph node tissue from children with FUO may serve as a efficient tool for distinguishing infectious diseases from other diseases. This is especially useful when a diagnosis cannot be determined with traditional methods.
3. mNGS targeted treatment can be administered in a timely manner and some underlying diseases can be indicated.

## Introduction

The diagnosis of fever of unknown origin (FUO) was first defined by Petersdorf in 1961.^1^ FUO etiologies mainly include infectious diseases, non-infectious inflammatory diseases, neoplastic diseases miscellaneous diseases and undiagnosed diseases.^2,3^ To date, there are no established standard diagnostic criteria for FUO in children. In recent studies, however, FUO in children has often been defined as unexplained fever that continues for 1 or 2 weeks.^4–6^ Recommendations for FUO evaluation involve an elaborate history and physical examination, routine laboratory tests, supplemental detection methods, radiographic examinations, and pathological examination. As mentioned above, physical examination is crucial for the diagnosis of FUO. Lymphadenopathy, which is defined as an abnormal lymph node size and/or characteristics, usually accompanies FUO and is more common in children, with 44% of FUO patients under 5 years old developing lymphadenopathy.^7^ Notably, new techniques have been applied in diagnosing children with FUO, for example, FDG-PET serves as a useful tool.^8,9^ M.-H. Bentza’s research noted that FUO with lymphadenopathy should be considered a separate entity that mainly originates from infection and malignancy, and the diagnosis relies on biopsy examination.^10,11^ Considering these factors, biopsy of lymph nodes in children with FUO may help clinicians reach a final diagnosis. However, the extent to which lymphadenopathy contributes to FUO remains unknown. With the recent progress in the development of techniques for the detection of pathogens, studies have shown that the rate of pathogen positivity according to metagenomic next-generation sequencing (mNGS) is much greater than that according to traditional methods, with analyses based on tissue samples showing better performance.^12,13^ mNGS is an unbiased approach that theoretically detects all kinds of pathogens in a specimen and is especially suitable for rare pathogens. This technology is becoming increasingly applicable in clinical work due to its high sensitivity, rapid speed, and cost-effectiveness.^14^ Therefore, we compared differences between different kinds of diseases to identify clinical clues aiding in diagnosis. We performed pathogen testing of lymph node tissue by mNGS for some patients whose diagnosis was difficult to determine to contribute to the understanding of FUO diagnosis in children with lymphadenopathy revealing how much assistance mNGS could provide in clinical management.

## Methods

### Patient study

A total of 387 patients with FUO accompanied by lymphadenectasis were retrospectively reviewed from January 1, 2020, to December 31, 2021, at Shanghai Children’s Medical Center (SCMC), China. Patients who underwent biopsy examinations were enrolled according to the following inclusion criteria:

Main criteria:

Fever >38.3 °C forå 1 week (empirical antibiotics for at least 2 days if necessary);

No diagnosis after medical history and routine examinations (routine assessments of blood, CRP, PCT, pulmonary imaging, abdominal ultrasonography, ultrasonic cardiogram, etc.).

Auxiliary criteria:

1. Weight loss greater than 10% in the past six months
2. More than 2 regional lymphadenectases
3. Lymph nodes larger than 2.0 cm
4. Enlargement of the supraclavicular lymph nodes
5. Hard and fixed lymph nodes
6. Hepatosplenomegaly

The exclusion criteria were as follows:

1. Traditional methods showed positive results, which were considered associated with the current clinical presentation
2. Fever decreased or clinical situation improved after empiric treatment
3. The guardian refused a lymph node biopsy

**Fig. 1:**
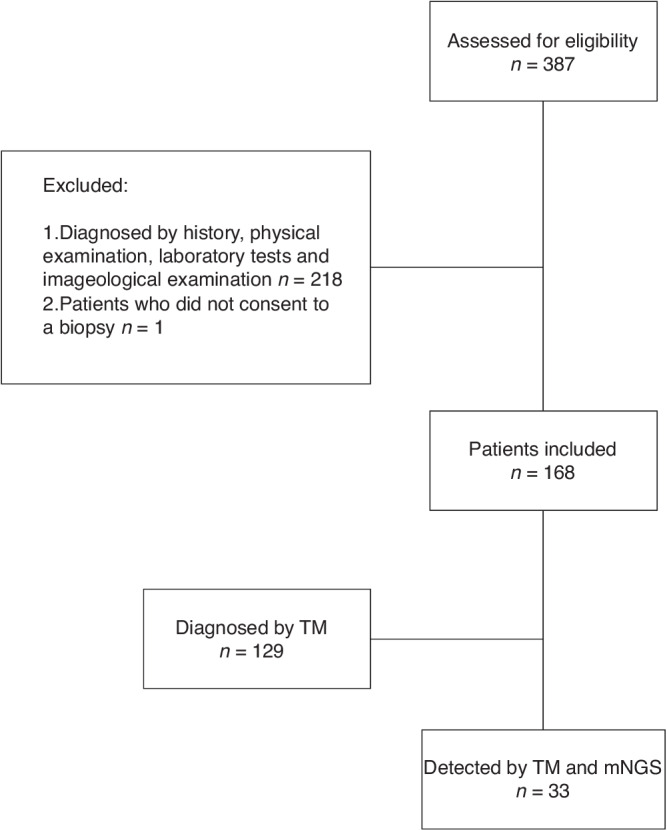
Flowchart of the present study. Flow diagram illustrating the design of the present study.

Patients who met the main criteria and had at least one auxiliary criterion were included. A total of 168 patients were enrolled during the study period. The study design is illustrated in Fig. 1.

Lymph node tissues were assessed by mNGS when the etiology was unclear and an infectious disease was strongly suspected. The final diagnoses were determined by combining clinical manifestations, microbiological testing, and pathological diagnosis. The study was approved by the Institutional Review Board of Shanghai Children’s Medical Center (SCMCIRB-K2017070), and written informed consent was obtained from the parents of each patient.

### Disease groups associated with FUO

All patients enrolled were divided into the following five groups according to the final diagnosis, which was based on the pathology results and clinical manifestations as well as laboratory test and imaging examination results: infectious group, non-infectious inflammatory diseases, neoplastic diseases, miscellaneous diseases, and undiagnosed diseases The clinical characteristics of the different disease groups were compared.

### Histopathology

Hematoxylin-eosin (HE), periodic acid-Schiff (PAS) and acid-fast staining were performed on paraffin sections. Immunostaining was conducted by EnVision, and antibodies against ALK, BCL-2, BCL-6, LCA, Lys and others (Long Island Biotechnology, Shanghai, China) were used. EBER in situ hybridization was also performed for all patients (BOND Ready-to-Use ISH EBER Probe, Leica, Wetzlar, Germany).

### Process of mNGS

#### Sample processing and DNA extraction

mNGS was conducted on samples from 33 patients. For metaDNA-seq, DNA was extracted from samples using a QIAamp® UCP Pathogen DNA Kit (Qiagen) according to the manufacturer’s instructions.^15^ For metaRNA-seq, RNA was extracted using a QIAamp® Viral RNA Kit (Qiagen), and ribosomal RNA was removed with a Ribo-Zero rRNA Removal Kit (Illumina). cDNA was generated by using reverse transcriptase and deoxynucleotide triphosphates (Thermo Fisher).

#### DNA library preparation and sequencing

Libraries were established for DNA and cDNA samples using a Nextera XT DNA Library Prep Kit (Illumina).^16^ Next, the library pools were loaded onto an Illumina NextseqCN500 sequencer for 75 cycles of single-end sequencing to generate approximately 20 million reads for each library.

#### Analysis of sequencing data

Low-quality reads, adapter contaminants, duplicate reads and reads shorter than 50 bp were removed using Trimmomatic.^17^ Low-complexity reads were removed using Kcomplexity with default parameters. Human sequence data were identified and excluded using Burrows‒Wheeler Aligner software.^18^ One human (hg38), 26207 bacterial, 308 archaeal, 54 fungal, 9021 viral, 178 invertebrate, and 39 protozoan reference genomes were downloaded from the National Center for Biotechnology Information (https://ftp.ncbi.nlm.nih.gov/genomes/).

### Traditional pathogen investigations

Blood samples were collected for bacterial culture, serological EBV and CMV antibody detection, *Epstein‒Barr virus* (EBV) and *cytomegalovirus* (CMV) PCR detection, and interferon gamma release assay (IGRA), beta glucan (G) test, and galactomannan (GM) tests. For patients with respiratory symptoms, nasopharyngeal swabs or sputum specimens were collected for a multiple respiratory PCR set in which *adenovirus* (ADV)*, influenza A, influenza B, respiratory syncytial virus* (RSV)*, parainfluenza virus* and *Mycoplasma pneumoniae* (MP) were detected. Traditional methods (TMs) included bacterial culture, serological EBV and CMV antibody detection, EBV-DNA, CMV-DNA, and IGRA assessment, G test, GM test and the respiratory pathogen test mentioned above.

### Statistical analysis

In this study, a *P* value of <0.05 was considered statistically significant. Categorical variables are expressed as frequencies and percentages. The chi-square test was used to compare groups. Continuous variables are expressed as the means and standard deviations, and comparisons were conducted using Student’s *t* tests. Variables that had a *P* value < 0.05 in univariate logistic analysis were included in multivariate logistic regression analysis. Multivariate analysis using stepwise forward selection was applied to create a logistic proportional hazards model to determine the predictive value for infectious diseases. The inclusion criterion for the factors was *P* < 0.05. The sensitivity and specificity of the model were evaluated using receiver operating characteristic (ROC) curve analysis. Analyses were performed using SPSS v22.0.

## Results

### General characteristics of the patients

During the study period, 168 children with a fever longer than 7 days and lymphadenopathy were enrolled in our study according to the inclusion criteria mentioned above, and all the children underwent pathological examinations. The diagnoses of thirty-three patients were difficult to confirm based on clinical manifestations, laboratory examinations and imaging examination, and the patients further underwent mNGS detection using abnormal lymph node tissue samples. The clinical manifestations and disease distributions of all 168 patients are shown in Table 1 and Table 2.

**Table 1:** Diseases distribution of the patients.

Characteristic	No (%)
Total	168
Age (y)	8.00 ± 4.22
Sex, no. (%)	
Male	103(61.31)
Female	65(38.69)
Symptoms and signs	
Duration of fever (d)	16.435 ± 8.426
Weight loss, no. (%)	18(10.71)
More than 2 regional lymphadenectases, no. (%)	61(36.31)
Node located in the supraclavicular region, no. (%)	11(6.55)
Hepatosplenomegaly, no. (%)	37(22.02)
Arthralgia, no. (%)	10(5.95)
Diameter of lymph nodes (cm)	2.251 ± 0.733
Prognosis after treatment, no. (%)	
Surviving	166(98.81)
Nonsurviving	2(1.19)

Data are no. (%) of patients, mean ± standard deviation (SD).

**Table 2:** Diagnosis and disease distribution of the patients.

Diagnosis	No. (%)
Infectious diseases	
Virus infection	25(14.88)
Bacterial infection	28(16.67)
Fungal infection	1(0.60)
Other rare pathogen infection	1(0.60)
Non-Infectious inflammatory diseases	
Autoimmune disease^a^	12(7.14)
Rheumatic	11(6.55)
Neoplastic diseases	
Lymphoma	5(2.98)
Leukemia	1(0.60)
Solid tumor	1(0.60)
Post transplantation lymphoproliferation disease	4(2.38)
Miscellaneous diseases	
Subacute necrotizing lymphadenitis	64(38.10)
Kawasaki disease	7(4.17)
Langerhans cell histiocytosis	2(1.19)
Undiagnosed diseases	6(3.57)

Data are no. (%) of patients, mean ± standard deviation (SD).

^a^Autoimmune diseases include 6 systemic lupus erythematosus, 5 unclassified connective tissue disease and 1 sicca syndrome.

### Clinical characteristics of different disease groups

Different disease groups exhibited unique manifestations (6 undiagnosed patients were not compared). Patients in the infectious disease group were the youngest, and the oldest were in the miscellaneous diseases group. Weight loss was more likely to be observed in the neoplastic group and was also commonly observed in the non-infectious inflammatory diseases group. As expected, hepatosplenomegaly was the most common complication in the neoplastic group. There were no significant differences in the diameter of the lymph nodes or the presence of more than 2 regional lymphadenectasis among the groups. If lymphadenectasis was discovered in the supraclavicular region, there was a greater likelihood of the final diagnosis being neoplastic diseases. However, if arthralgia was present, a non-infectious inflammatory disease was the most likely diagnosis. The duration of fever was longer for patients with non-infectious inflammatory diseases and neoplastic diseases. With further investigation, differences were observed not only in terms of general characteristics and symptoms but also in some laboratory test results. More than two types of hemocytopenia were more likely to be observed in the neoplastic diseases group, as expected. Interestingly, the WBC count was greater in the neoplastic group than in the infectious disease group. Furthermore, the lowest WBC count was observed in the miscellaneous disease group and the neoplastic disease group. As expected, the hemoglobin level was lowest in the neoplastic disease group. The LDH level was extremely high in the neoplastic disease group. The CRP level in the infectious disease group was even lower than that in the non-infectious inflammatory disease group, which was unexpected. The ESR was highest in patients in the non-infectious inflammatory disease group, followed by patients in the neoplastic disease group. For PCT and Lac, no significant differences were observed among the four groups (Table 3). According to our multivariable analysis, hepatosplenomegaly and the LDH level were associated with infectious disease (OR 10.559 and 0.998; 95% CI 2.172–51.336 and 0.997–1.000; *P* = 0.003 and *P* = 0.020). Arthralgia was correlated with non-infectious inflammatory disease (OR 29.391; 95% CI 2.307–374.459; *P* = 0.009). Weight loss and a node located in the supraclavicular region may indicate neoplastic disease (OR 918.889 and 4380.842; 95% CI 1.393–606193.104 and 2.630–9456316.108; *P* = 0.039 and *P* = 0.032) (Table 4).

**Table 3:** Comparison of clinical features between different disease groups.

Characteristics	Infectious diseases (*n* = 55)	Non-infectious inflammatory diseases (*n* = 23)	Neoplastic diseases (*n* = 12)	Miscellaneous diseases (*n* = 72)	SE	95%CI	P value
Age (y)	6.53 ± 4.49	8.26 ± 4.16	7.03 ± 5.24	9.31 ± 3.44	0.331	7.39–8.70	0.002
Weight loss	4(7.30)	5(21.74)	3(25.00)	6(8.33)	0.025	0.07–0.17	0.037
Over 2 regional lymph node enlargement	22(40.0)	8(34.00)	7(58.30)	22(30.56)	0.038	0.29–0.44	0.274
Hepatosplenomegaly	20(36.40)	3(13.00)	5(41.70)	8(11.11)	0.033	0.16–0.29	0.002
Diameter of lymph node(by ultrasound)	2.16 ± 0.74	2.19 ± 0.62	2.08 ± 0.43	2.302 ± 0.82	0.064	2.101–2.353	0.710
Node located in supraclavicular	1(1.80)	0(0.00)	6(50.00)	4(5.55)	0.020	0.03–0.11	<0.001
Arthralgia	0(0.00)	8(34.80)	2(16.70)	0(0.00)	0.019	0.02–0.10	<0.001
More than two types of hemocytopenia	8(14.50)	0(0.00)	4(33.30)	5(6.944)	0.024	0.06–0.15	0.010
Duration of fever	15.40 ± 7.64	20.49 ± 10.18	19.58 ± 16.46	15.35 ± 5.65	0.659	15.11–17.71	0.027
WBC (*10^9/L)	7.72(4.76,11.57)	6.74(4.34,10.80)	10.99(4.95,19.80)	5.02(	0.540	6.411–8.512	<0.001
Hb(g/L)	107.46 ± 22.96	108.65 ± 20.85	98.01 ± 24.52	117.48 ± 18.55	1.700	108.049–114.761	0.005
Neutrophil percentage (%)	50.10 ± 18.45	59.37 ± 15.86	54.51 ± 20.20	47.48 ± 13.46	1.301	47.976–53.115	0.019
Platelet (*10^9/L)	213.00(145.75,320.50)	284.50(197.00,402.00)	271.00(69.750,39.50)	221.00(166.20,309.50)	10.780	224.11–266.69	0.010
LDH (U/L)	635.50(519.00,1004.00)	691.00(518.00,1155.50)	1275.50(699.00,2232.00)	782.00(547.00,1292.00)	89.115	938.02–1290.26	0.021
CRP (mg/l)	12.00(2.55,49.75)	35.5.(11.00,77.00)	45.00(18.25,98.25)	10.00(2.70,34.50)	3.789	23.712–38.686	<0.001
ESR (mm/h)	38.71 ± 27.27	62.17 ± 36.10	50.22 ± 33.10	39.79 ± 25.21	2.446	38.895–48.656	0.005
PCT (ng/mL)	0.25(0.08,5.01)	0.25(0.09,0.45)	1.24(0.23,6.33)	0.25(0.08,0.47)	1.383	2.823–8.291	0.049
Lac(mmol/L)	1.75 ± 1.13	1.82 ± 0.62	2.28 ± 2.22	1.44 ± 0.69	0.115	1.418–1.876	0.319

**Table 4:** Multivariable analysis of clinical features related to different diseases group

	Characteristics	OR	95% CI	*P* value
Infectious diseases	Hepatosplenomegaly	10.559	2.172–51.336	0.003
	LDH (U/L)	0.998	0.997–1.000	0.020
Non-infectious inflammatory diseases	Arthralgia	29.391	2.307–374.459	0.009
Neoplastic diseases	Weight loss	918.889	1.393–606193.104	0.039
	Node located in supraclavicular	4380.842	2.630–9456316.108	0.032
Miscellaneous diseases	Hepatosplenomegaly	0.18	0.048–0.679	0.011

### Pathogen distribution in the infectious disease group detected by mNGS and traditional methods

Among all 168 patients, identifying the cause of disease through clinical manifestations and traditional routine examinations was difficult in 33 patients, and conditions did not improve after empirical treatment. To determine the cause, we carried out pathological examination of the lymph node tissue as well as mNGS. The pathogen distributions are shown in Table 5. One patient after hematopoietic stem cell transplantation was EBV positive according to mNGS, and EBV-DNA in plasma was diagnosed post-transplantation lymphoproliferation according to pathology. The other 7 patients with EBV-positive lymph node tissue showed negative results by qPCR of PBMC samples. One of those patients was diagnosed with hemophagocytic syndrome, which was mainly considered to be driven by EBV infection, 2 patients were diagnosed with necrotizing lymphadenitis, and 4 patients were diagnosed with acute lymphadenitis. The overall positive rates for mNGS and traditional methods were 60.60% and 43.63%, respectively. Three *Mycobacterium bovis* infections and one *Mycobacterium abscessus* infection were detected by mNGS but could not be distinguished by traditional methods. Three patients with *Mycobacterium bovis* infection were diagnosed with Bacilli Calmette-Guerin disease. All three patients were under 1 year old and were further confirmed to have immunodeficiency diseases by whole-exome sequencing. Considering the underlying disease and the lack of improvement in clinical conditions after treatment, a patient whose blood culture showed positive results also underwent biopsy Pathology ultimately revealed that the patient with aplastic anemia who underwent hematopoietic stem cell transplantation was diagnosed with lymphoma.

**Table 5:** Pathogens detected in the infectious disease group by different methods.

Pathogens	Number	
	mNGS (*n* = 33)	TM (*n* = 55)
Virus	10	17
EBV	8	15^a^
CMV	/	2 ^a^
Parvovirus B19	1	/
Torque teno virus	1	/
Bacteria	8	2^b^
*Acinetobacter baumannii*	1	/
*Staphylococcus aureus*	2	/
*Streptococcus pneumoniae*	1	/
*Mycobacterium abscess*	1	/
*Mycobacterium bovis*	3	/
*Stenotrophomonas maltophilia*	0	1
*Streptococcus mitis*	0	1
Fungi	1	5^c^
*Penicillium marneffei*	1	/
Cryptococcus	0	2^d^
Other rare pathogens	1	/
*Coxiella burnetii*	1	/
Total (%)	60.60	43.63

^a^EBV-DNA > 500 copies/mL and CMV-DNA > 500 copies/mL in plasma.

^b^Blood culture positive.

^c^1 GM test positive and 4 G test positive.

^d^Blood culture positive.

### Comparison of mNGS and traditional methods for pathogen detection

**Fig. 2:**
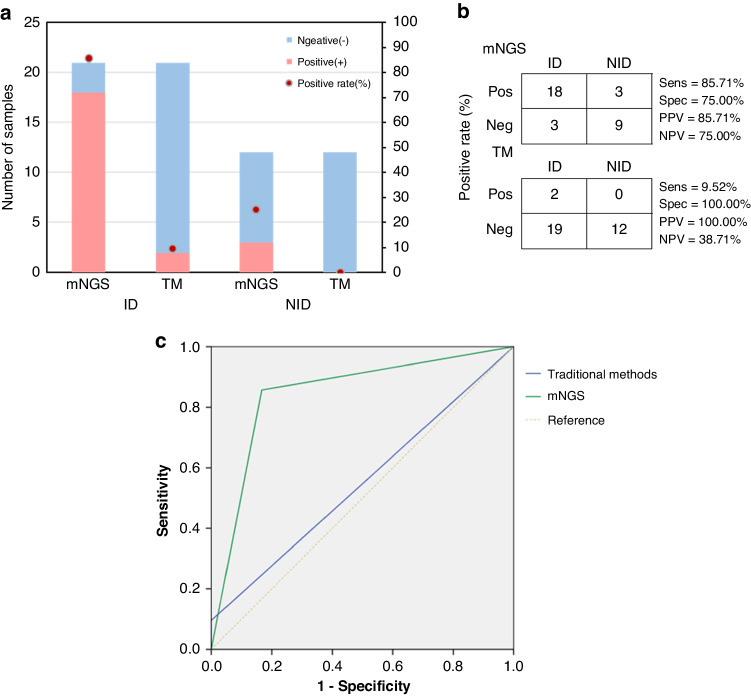
Comparison of mNGS and traditional methods for pathogen detection. **A** The positive rates of mNGS and traditional methods for ID and NID. **B** 2 × 2 cross tables comparing the performance of mNGS and traditional methods for distinguishing ID from NID. **C** Receiver operating characteristic (ROC) curve analysis showing the power of mNGS for diagnosing infectious lymphadenopathy. The area under the ROC curve (AUC) for mNGS was 0.845 (95% CI 0.694–0.997; *P* = 0.001), and that for traditional methods was 0.548 (95% CI 0.346–0.750; *P* = 0.653).

We compared the positive rate between mNGS and traditional methods for infectious disease (ID) and non-infectious disease (NID) (Fig. 2A). The results showed that the positive rate of mNGS in the ID group was much higher than that for traditional methods (85.71% vs. 9.52%; *P* < 0.001). mNGS exhibited approximately 76.19% higher sensitivity than traditional methods (85.71% vs. 9.52%; *P* < 0.001), although the specificity of mNGS was slightly lower than that of traditional methods, with no statistical significance (75.00% vs. 100.00%; *P* = 0.729). The PPV and NPV for identifying ID and NID by mNGS were calculated to be 85.71% and 75.00%, respectively, while those for the traditional methods were 100% and 38.71%, respectively (Fig. 2B). The diagnostic value of mNGS and traditional methods was also assessed by receiver operating characteristic (ROC) curve analysis, which indecated that mNGS had a greater diagnostic accuracy, with an area under the curve (AUC) of 0.845 (95% CI 0.694–0.997; *P* = 0.001) (Fig. 2C).

**Fig. 3:**
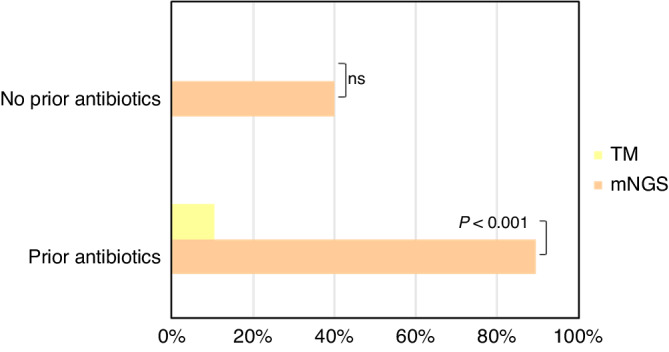
Comparison of positive rates between mNGS and traditional methods after prior antibiotic exposure. A significantly higher positive rate was detdcted for mNGS than for traditional methods for samples from patients with prior antibiotic exposure (*P* < 0.001), suggesting that mNGS is less affected by prior antibiotic exposure.

Furthermore, we investigated whether prior antibiotic exposure affects the results of pathogen detection using mNGS. In this study, 28 patients were given antibiotics prior to mNGS and culture detection. Nineteen of these 28 children were divided into the ID group, with 17 positive results according to mNGS and 2 positive results according to traditional methods (89.47% vs. 10.53%, *P* < 0.001) (Fig. 3). In addition, according to the pathogen results we obtained, treatment adjustments were made for 24 (72.72%) patients, and administration of antibiotics was discontinued or reduced in 18 (54.54%) patients.

## Discussion

In the present study, we investigated discrepancies in the clinical manifestations of different kind of diseases in children who had both an unresolved fever for more than 1 week and lymphadenopathy. A total of 168 patients were enrolled in our study. Considering the prolonged fever resistance and the necessity of clearly determining the etiology of their disease because previous treatments were unsuccessful, pathological examinations were carried out on abnormal lymph nodes. In our study, diagnoses were determined from clinical manifestations, laboratory tests, radiographic examinations, and pathological examination, which is the gold standard. Patients were divided into five different disease groups: infectious diseases, non-infectious inflammatory diseases, neoplastic diseases, miscellaneous diseases, and undiagnosed diseases. Six undiagnosed patients were eliminated when comparing the differences in clinical manifestations. Subacute necrotizing lymphadenitis accounted for the largest proportion of cases, and acute bacterial lymphadenitis was the most common infectious disease, followed by viral infection. In Elisabetta Venturini’s research,^19^ EBV and nontuberculous mycobacteria were the most common etiological agents of lymphadenopathy, whereas only half of the patients reached a specific diagnosis through the use of one or more methods; serological, microbiological, biomolecular or histological investigations; or even surgical sample analyses. Our inclusion criteria may have led to the largest proportion of patients having necrotizing lymphadenitis, although infectious diseases also accounted for a relatively large proportion of the patients.

When comparing differences among the four groups, each group showed some unique manifestations, as we hypothesized; however, some results were unexpected as well. Patients in the infectious disease group were the youngest, and the oldest patients were in the miscellaneous group. Lymphadenopathy is more common in children than in adults. Kimia et al.’s study^20^ revealed that age ≤3 years was independently associated with suppurative adenitis among children presenting with cervical adenopathy. Previous research^21^ has shown that younger age is associated with a greater risk of suppurative lymphadenitis, but in the present study, the younger age of the infectious disease group was mainly attributed to the greater degree of EBV infection. Among the miscellaneous disease group, subacute necrotizing lymphadenitis, which is also called Kikuchi-Fujimoto disease, accounted for a large proportion of cases; this disease usually affects young Asian adults, contributing to the older age of these individuals.^22^ The median age of patents with this disease is reported to be 11.2 years.^23^ Weight loss is often associated with neoplastic diseases. In our present study, weight loss was more likely to occur in the neoplastic disease group, with non-infectious inflammatory diseases accounting for a large proportion of cases involving weight loss. Recent studies have shown that BMI and weight loss are predictors of cause-specific mortality and worsening disability in rheumatoid arthritis patients.^24,25^ Arthralgia was found in not only patients with immune disorders but also those with neoplastic diseases in our study. Hepatosplenomegaly, involving a lymph node located in the supraclavicular region, is usually found in patients with neoplastic diseases and provides some value in distinguishing this kind of disease from others. Furthermore, differences in routine laboratory tests results among the four disease groups were observed. We observed that hemocytopenia often occurrs in patients with neoplastic diseases, although the lowest WBC count was found in the miscellaneous disease group, which may be attributed to the large proportion of subacute necrotizing lymphadenitis patients. Unexpectedly, the CRP concentration and ESR were greater in patients with neoplastic diseases and non-infectious inflammatory diseases, han in patients with infectious diseases, possibly because of the large proportion of patients with EBV infection in our study, a disease rarely associated with high CRP concentration or ESR. According to Gosche’s research, the CRP level and WBC count showed no significance, regardless of infectious diseases or otherwise,^26^ and significantly elevated LDH levels are associated with malignancy.^10,27^ In our research, the LDH level was extremely high, and may serve as a useful indicator of neoplastic diseases. The diameter of the lymph node (by ultrasound) was not significantly different among the different disease groups. Multivariate analysis was also performed. Hepatosplenomegaly and lower LDH levels were confirmed to be associated with infectious diseases. Arthralgia was correlated with non-infectious inflammatory diseases. Weight loss and a node located in supraclavicular region indicated neoplastic diseases. In summary, clinical manifestations and routine laboratory test results vary among different kinds of diseases; only limited clues can be drawn from these different clinical characteristics, and the final diagnosis is usually unachievable without biopsy examination, suggesting that treatment is thus usually empirical by clinicians. With such prolonged fever and many other complaints, identifying the actual pathogenesis and administering targeted therapy, which can ameliorate concerns among children and their families, are important.

As mentioned above, indicators of infectious diseases have rarely been confirmed. Despite the combined use of clinical manifestations and routine test results, conclusions were not reached for 33 patients. Studies^12,13,28^ have demonstrated that mNGS has higher sensitivity for pathogen identification; in addition, the application of mNGS for FUO in adults has significantly greater diagnostic efficacy than conventional methods.^29^ With improvements in new technology, the main infectious agents in lymphadenopathy that can be detected have changed. For example, with PCR methods, *Bartonella henselae*, which is responsible for cat scratch disease, and many other agents that are difficult to detect by traditional methods can be identified and should be considered.^30,31^ For further investigation, 33 lymph node tissues were assessed with mNGS in our study; 20 of these specimens were positive, providing a guide for effective treatment. In our research, EBV infection was the only positive result detected by traditional methods in the 33 patients who underwent mNGS. Seven patients with EBV-positive lymph node tissue showed negative results according to qPCR of PBMC samples. However, the importance of EBV positivity by mNGS in tissue samples has not been determined. In our research, 1 patient was diagnosed with hemophagocytic syndrome, which was mainly considered to be caused by EBV infection. Three patients under 1 year old with *Mycobacterium bovis* infection were diagnosed with Bacilli Calmette-Guerin disease. In addition, immunodeficiency diseases in these three patients were further confirmed by whole exome sequencing. One was an IL2RB1 gene mutation that leads to susceptibility to tuberculosis, and the other was a JAK3 gene mutation that is associated with severe combined immunodeficiency and a STAT1 gene mutation that also increases susceptibility to tuberculosis. In addition to the superiority of strain identification, mNGS also showed advantages for rare pathogen detection. For example, *Coxiella burnetii* and *Penicillium marneffei* were only revealed in our study by mNGS. When comparing the positivity rates of mNGS and traditional methods, the high performance of the former helped us to identify the cause of infectious disease undetectable by traditional methods. In addition, the types of specimens, usually blood samples, used for traditional tests are limited, and it may be difficult to discover local infections. Lymph node tissue, which cannotbe evaluated by using traditional methods, was used in our study for mNGS detection and was superior for detection local infections. Miao et al.’s study^13^ revealed that tissue samples have significantly greater sensitivity for mNGS detection than for culture detection. Yue Tao’s research^12^ showed that tissue samples detected by mNGS yielded a 62.1% positive rate, which was greater than that of blood samples. Considering the difficulty of obtaining tissue samples, a highly sensitive pathogen detection method is crucial. In addition, Li et al.’s study^32^ revealed that mNGS combined with computational analysis can identify pathogens in formalin-fixed corneal specimens. In the present study, two unstained sections of lymph node tissues detected by mNGS were positive. According to our clinical experience, a positive result may be more likely if mNGS detection can be carried out on the abnormal part of pathological samples. Briefly, pathogen detection using infectious tissue samples by mNGS has the advantages of a high positive rate for local infection and strain identification when compared with traditional methods. Therefore, lymph node tissue testing by mNGS is a reasonable choice for identifying the etiology of FUO with lymphadenopathy.

Antibiotic exposure usually has some influence on the results of culture tests. Previous stydies^12,13,33,34^ have shown that the sensitivity of mNGS for identifying pathogens is minially affected by prior antibiotic exposure, and we drew a similar conclusion compared to culture methods. In addition, with the pathogen results we obtained, treatment adjustments were made in 3/4 of the patients, and reductions in antibiotic administration were carried out in more than 1/2 of the patients. This approach reduced the rational use of antibiotics and improved the patients’ conditions.

Nevertheless our study has several limitations. First, this was a single-center study, and the composition of children with lymphadenopathy and FUO might be biased. Second, considering the difficulty of obtaining lymph node tissue cultures and because no abscess samples was obtained, we conducted a culture test using blood samples, which may have led to a lower positive rate when the infection was local. Third, the importance of EBV positivity by mNGS in tissue samples remains unclear, although a patient with HLH, which considered to be driven by EBV infection, was diagnosed in our research, this finding needs further investigation.

In summary, our study revealed that subacute necrotizing lymphadenitis is the most common disease in children with lymphadenopathy and FUO, followed by viral infection and acute bacterial lymphadenitis. Different kinds of diseases present different characteristics, and some clinical manifestations help pediatricians differentiate diagnoses. If the diagnosis is unclear and empirical treatment does not improve the patient’s conditions, mNGS performed on lymph node tissue rather than traditional pathogen detection methods is a useful tool for distinguishing infectious diseases from others and identifying certain causative agents, suggesting an underlying disease condition, guiding pediatricians toward targeted therapy, and ultimately benefiting patients.

## Data Availability

The datasets generated during and/or analyzed during the current study are available from the corresponding author upon reasonable request.
